# Disparities in the use of telehealth at the onset of the COVID-19 public health emergency

**DOI:** 10.1177/1357633X20963893

**Published:** 2023-01

**Authors:** Robert P Pierce, James J Stevermer

**Affiliations:** Department of Family and Community Medicine, University of Missouri, USA

**Keywords:** Digital divide, equity, telehealth, health disparities, COVID-19, pandemic

## Abstract

**Introduction:**

The coronavirus disease 2019 (COVID-19) pandemic resulted in an unprecedented expansion in telehealth, but little is known about differential use of telehealth according to demographics, rurality, or insurance status.

**Methods:**

We performed a cross-sectional analysis of 7742 family medicine encounters at a single USA institution in the initial month of the COVID-19 public health emergency (PHE). We compared the demographics of those using telehealth during the PHE to those with face-to-face visits during the same time period; we also compared the demographics of those using full audio-video to those using audio-only.

**Results:**

The likelihood of any telehealth visit in the first 30 days of telehealth expansion was higher for women, those age 65 years and older, self-pay patients, and those with Medicaid and Medicare as primary payers. The likelihood of a telehealth visit was reduced for rural residence and Black or other races. Among all telehealth visits, the likelihood of a full audio-video telehealth visit was reduced for patients who were older, Black, from urban areas, or who were self-pay, Medicaid, or Medicare payer status.

**Discussion:**

Significant disparities exist in telehealth use during the COVID-19 PHE by age, race, residence and payer.

## Introduction

The worldwide outbreak of coronavirus disease 2019 (COVID-19) has disrupted the delivery of primary care in the USA. Under the National Emergency declaration,^1^ the Centers for Medicare and Medicaid Services (CMS) extended coverage eligibility for telehealth services,^2,3^ and telehealth regulatory requirements were eased.^4^ As a result, the provision of telehealth services has dramatically expanded during the COVID-19 pandemic.

Telehealth is effective and generally satisfies patients’ care expectations.^5–11^ Prior to the COVID-19 pandemic, the adoption of telehealth was slowed by payment constraints, provider concerns and organizational barriers.^12^ Additionally, disparities in access to and use of telehealth services existed across demographic and socioeconomic strata. For example, among insured patients, those who are employed and have post high-school education had higher uptake of telehealth services.^13^ Among residents of rural communities, patients who utilized telehealth visits were more likely to be white and non-Hispanic,^14^ younger,^15^ have insurance^16^ and live in a poorer community.^15^ Studies of telehealth associations with race and rurality had mixed results.^13,14,17^ This earlier research has evaluated telehealth use prior to the COVID-19 telehealth expansion, and little is known about telehealth use following the onset of the pandemic. To address these knowledge gaps, we conducted a cross-sectional analysis of primary care patients who utilized telehealth visits during the first 30 days after the expansion of telehealth services during the COVID-19 pandemic. We investigated potential disparities in telehealth use according to demographic (age, sex and race/ethnicity), rurality and insurance payer subgroups.

## Methods

### Study setting and population

We conducted an analysis based on telehealth utilization during the COVID-19 pandemic at a single academic centre in the USA (University of Missouri Health System, MU Health), the only academic medical centre in central Missouri, serving approximately 25 counties with a population of *c.* 800,000 people. The Department of Family and Community Medicine provides much of the primary care to the core of this region, with 89 clinicians based in seven outpatient practices across three different counties. Following the CMS announcement of expansion of telehealth under the national emergency 1135 waiver,^2^ MU Health implemented several procedural and administrative policies for telehealth visits, including (a) obtaining consent for telehealth; (b) conventions for titling medical documentation to reflect whether the visit was full telehealth (audio and video) or audio-only; (c) a requirement that providers document the duration of audio-only visits; and (d) a new distinct appointment type in the practice management software for telehealth encounters. This retrospective analysis included all finalized ambulatory clinic encounter documentation (‘Family Medicine Clinic Note’) completed by residents, attendings and advanced practice providers providing primary care in the Department of Family and Community Medicine. Encounter documentation during the first 30 days of the COVID-19 pandemic (17 March to 16 April 2020) was included. Visits with only operative notes were excluded. Scheduled visits to which the patient did not arrive and were not charged were excluded. Patient demographics were extracted directly from the MU Health Center electronic health record (EHR) database. Encounter data were determined based on administrative claims coding. Demographics were compared to similar population from the same practice sites and the same 30-day period in 2019.

### Patient and encounter classification

Encounters with charges for services associated with a telehealth appointment type were classified as telehealth.^18^ Encounters were classified as audio-only telehealth if charge data supported either virtual check-in (encounter codes G2010, G2012) or telephone evaluation and management visits (99441–99443). In some cases, charge data was incomplete due to delays in note completion. In these cases, authors determined visit type based on data extracted from individual charts and using the telehealth note titling and time documentation conventions established in health-system policy. Sex, race and ethnicity were self-reported. Insurance status was determined by primary payer listed on corresponding visit. Medicare Advantage plans were classified as private insurance. Rurality was assigned based on patient postal codes according to the Federal Office of Rural Health Policy.^19^

### Analysis

In order to determine how representative the study encounters were and how the demographics of patients compared to a non-pandemic year, demographics, rurality and payer status for patients with visits in the first 30 days of the COVID-19 telehealth expansion were compared to those with visits from the same 30-day period in 2019. Chi-square Student’s *t*-test with Welch’s approximation were used for comparisons where appropriate.

For all encounters during the COVID-19 telehealth expansion, likelihood of telehealth visits versus in-person encounters were determined using multivariate logistic regression models adjusted for sex, age (>18, 18–44, 45–64, and ≥65 years), race (white, Black or African American, other), ethnicity (Hispanic, non-Hispanic, other) rurality (rural or urban), payer status categories (private insurance, Medicaid, Medicare, or self-pay). For the telehealth encounters, likelihood of full audio-video telehealth encounters versus audio-only encounters was determined by estimating a multivariate regression model using the same variables. A *p*-value of 0.05 was considered statistically significant. Statistical analysis performed using Stata/IC v16.0 (College Station, TX). Patients with missing data were excluded from the logistic regression analysis. This study was reviewed by the University of Missouri Institutional Review Board and determined to be exempt, and informed consent was not separately obtained for this retrospective analysis.

## Results

**Figure 1.**
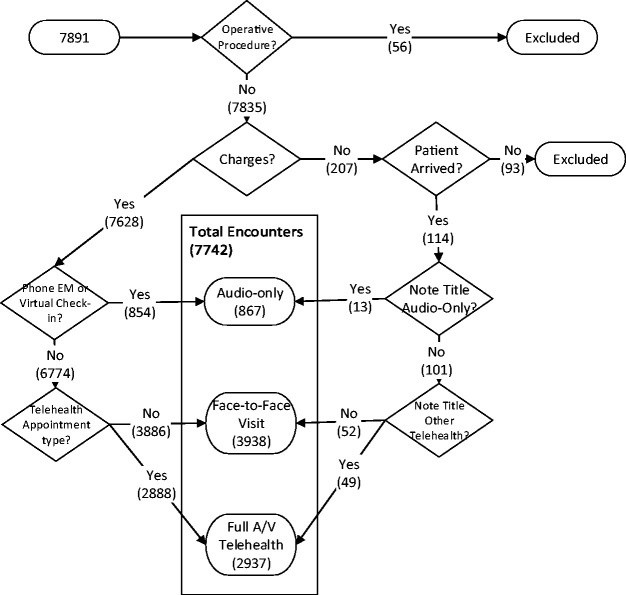
Classification of patients in the first 30 days of the COVID-19 public health emergency.EM: evaluation and management; A/V: audio/video.

The initial analysis included 7891 encounters during the first month of the COVID-19 telehealth expansion. Fifty-six were excluded as operative encounters, and 93 were excluded because the patient either never arrived or no charges were billed. Final analysis included 7742 encounters representing 6984 unique patients (Figure 1). Over the study period, there were 3938 face-to-face encounters and 3804 (49.1%) telehealth encounters. Of the telehealth encounters, 2937 (37.9%) were full audio-video and 867 (11.2%) were audio-only.

In the 2020 study period, there were 29.3% fewer total visits, including face-to-face visits, compared to the same period in 2019. The encounters in 2020 did not differ from those in the prior, non-pandemic year in mean age or percentage of patients from a rural postal code. However, we found slight differences by race, ethnicity, sex and payer. During the 2020 COVID-19 emergency, compared to the prior year, the proportion of encounters of any type increased for patients who were female (64.79% in 2020 v. 62.57% in 2019, *p* = 0.002), Black (10.66 v. 9.53%, *p* = 0.019), Hispanic (2.26 v. 1.75%, *p* = 0.012). The distribution of payers also differed between 2020 and the prior year, with a somewhat higher percentage of encounters during the COVID-19 emergency among patients with Medicaid and without insurance (Table 1).

**Table 1.** Demographics for family medicine patients seen in the first 30 days of the telehealth expansion at the University of Missouri compared to same period, prior year.

	Number of encounters 2020 COVID-19 (%)	Number of encounters 2019 (%)	*p-*value
Female	5016 (64.79)	6853 (62.57)	0.002
Mean age (years)	44.76 years	44.49 years	NS
Rural	2784 (36.00)	3867 (35.31)	NS
Hispanic or Latino or Spanish origin	175 (2.26)	191 (1.75)	0.012
Payer			<0.0001
Self- pay	483 (6.24)	373 (3.41)	
Medicaid	351 (4.53)	459 (4.19)	
Medicare	1074 (13.87)	1660 (15.16)	
Private insurance	5834 (75.36)	8461 (77.25)	
Race			0.019
White	6557 (84.69)	9358 (85.37)	
Black or African American	825 (10.66)	1045 (9.53)	
Other	360 (4.65)	559 (5.10)	
Total Encounters	7742	10953	

NS, not significant.

**Figure 2.**
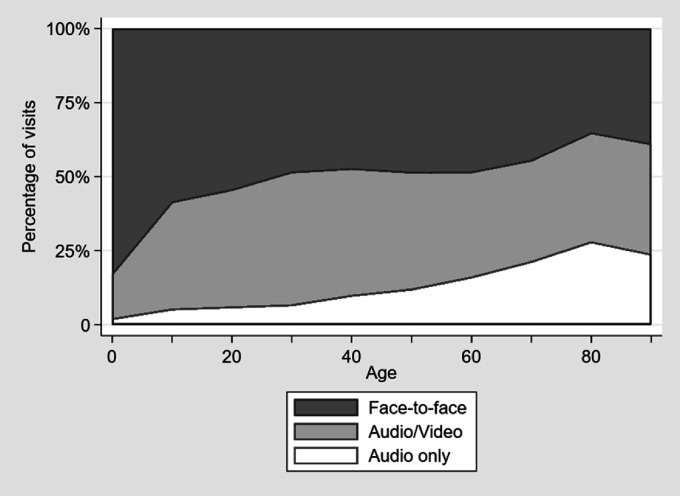
Probability of any telehealth encounter and audio-only telehealth encounter, by decade of age.

**Figure 3.**
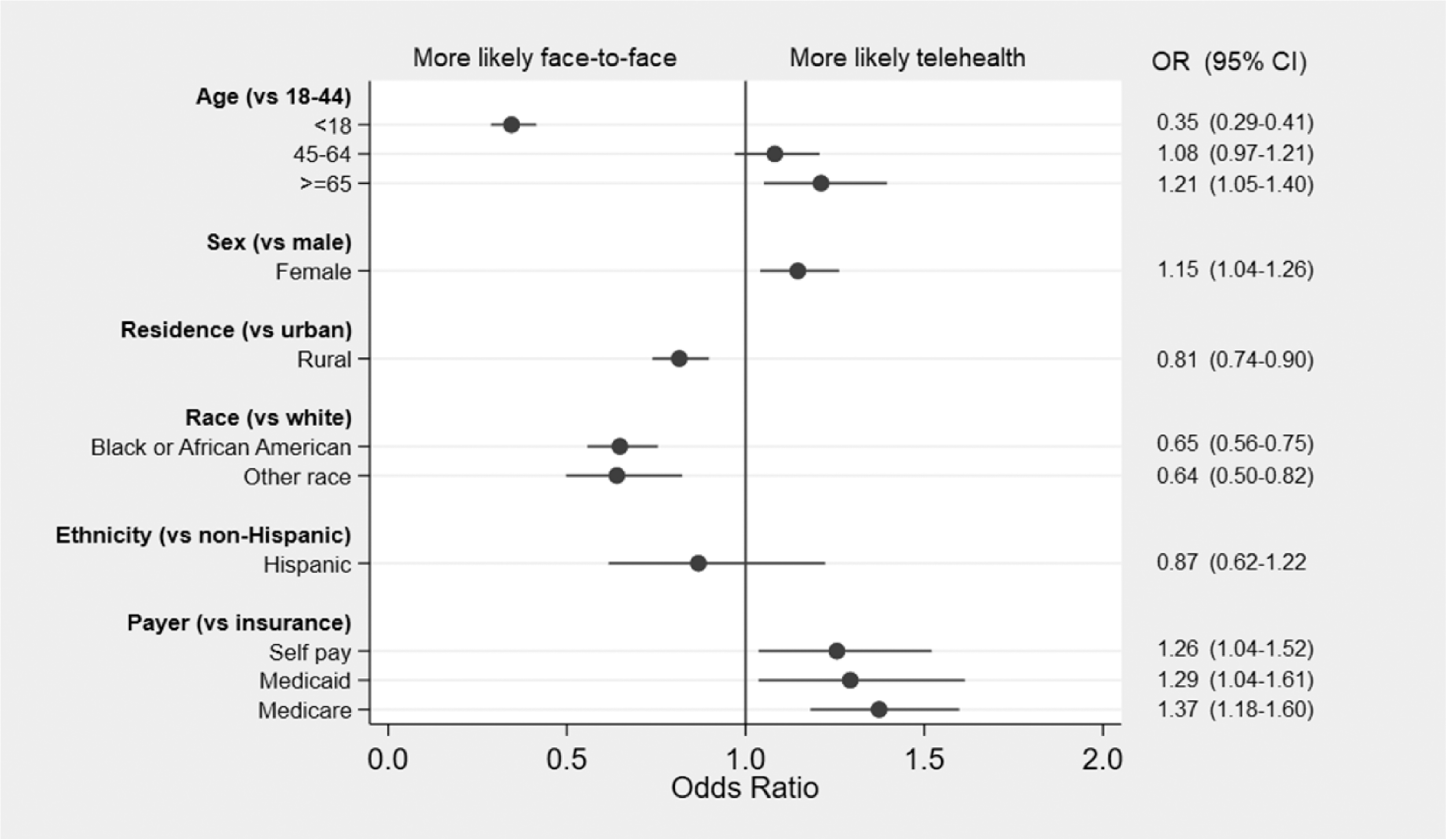
Odds ratios (ORs) for any telehealth visit (versus face-to-face) visit (multi-variate analysis).

The proportion of telehealth visits increased with increasing age (Figure 2). A total of 51 patients with missing ethnicity were excluded from the logistic regression analysis. In multivariate models, compared to those 18–44, the odds of any telehealth encounter were lower for those < 18 years (odds ratio (OR) 0.35, 95% confidence intervals (CI) 0.29–0.41) and higher for those 65 years and over (OR 1.21, 95% CI 1.05–1.40). Telehealth was more often used by women (OR 1.15, 95% CI 1.04–1.26) versus men and those with Medicare (OR 1.37, 95% CI 1.18–1.60), Medicaid (OR 1.29, 95% CI 1.04–1.61) and self-pay (1.26, 95% CI 1.04–1.52) compared with private insurance. Telehealth was used less often by Blacks (OR 0.65, 95% CI 0.56–0.75) and those identifying as other race (0.64, 95% CI 0.50–0.82), compared with whites, as well as those with rural residence (0.81, 95% CI 0.74–0.90) compared with urban (Figure 3).

**Figure 4.**
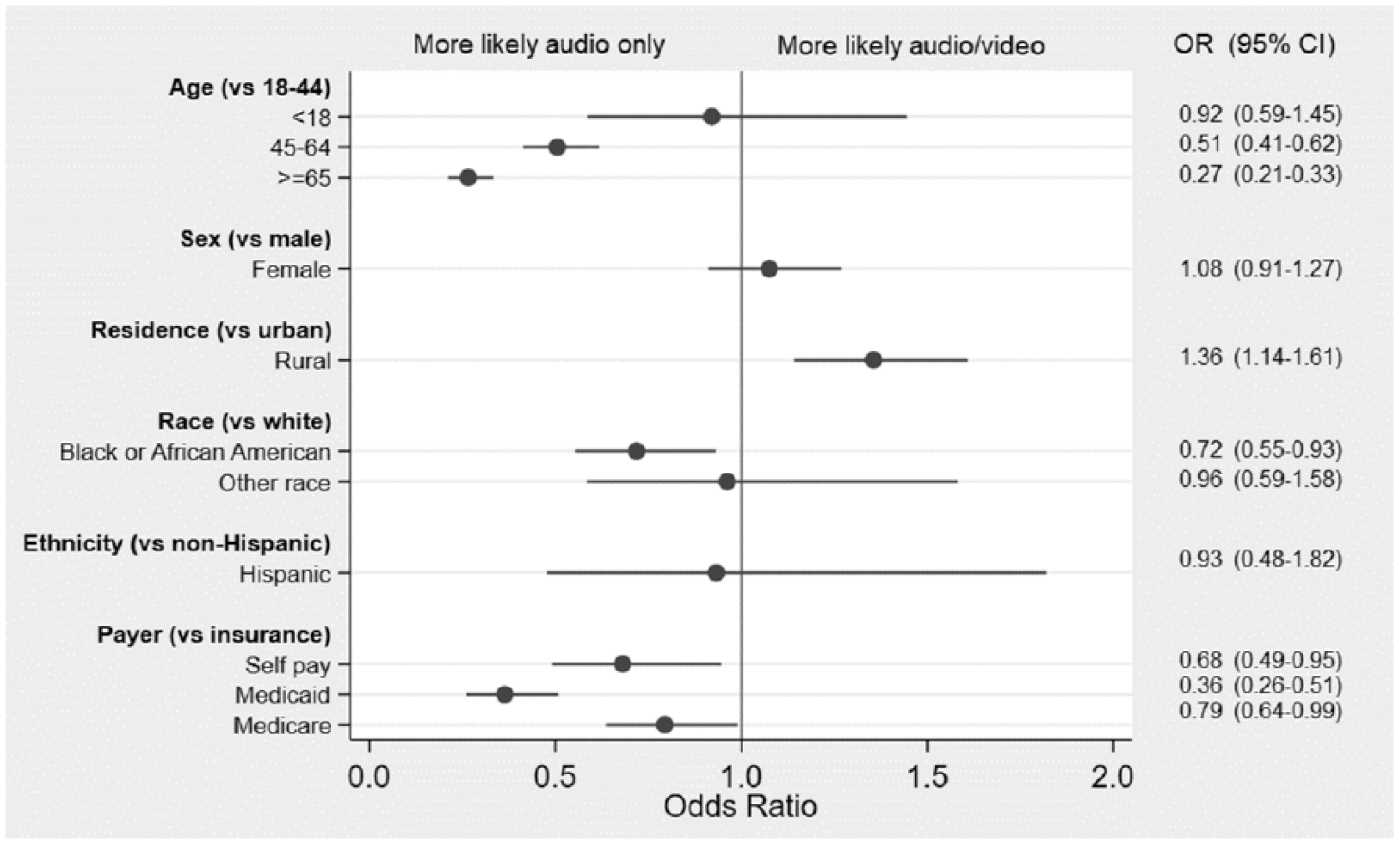
Odds ratios (ORs) for full audio-video (versus audio-only) telehealth visits. (multi-variate analysis).

In the analysis limited to telehealth encounters, the likelihood of having a full audio-video telehealth encounter (versus an audio-only encounter) was lower among those aged 45–64 (OR 0.51, 95% CI 0.41–0.62) and 65 years and older (OR 0.27, 95% CI 0.21–0.33). Full audio-video telehealth was also less likely among Blacks (OR 0.72, 95 CI 0.55–0.93) compared to whites and patients with Medicaid (OR 0.36, 95% CI 0.26–0.51), Medicare (OR 0.79, 95% CI 0.64–0.99), or self-pay (OR 0.68, 95% CI 0.49–0.95), compared to private insurance. Among the telehealth encounters, full audio-video was more common among those with rural residence (OR 1.36, 95% CI 1.14–1.61) compared with urban (Figure 4).

## Discussion

Our findings demonstrate several disparities in the use of telehealth during the COVID-19 telehealth expansion. Relative to the prior year, men, whites and non-Hispanics were less likely to be seen by any method, although the clinical significance of these small differences is unclear. During the COVID-19 public health emergency (PHE), telehealth visits were used more often by women, older patients, and those with Medicare, Medicaid, and self-pay status. Telehealth was used less often by those of non-white race and those from rural postal codes. Among those seen using telehealth, younger patients and those from a rural postal code were more likely to utilize full audio-video capability, and phone-only visits were more frequent with older patients, Blacks, and those with Medicaid, Medicare, and self-pay status. Ours is among the first studies to describe these disparities in telehealth use since the start of the COVID-19 pandemic.

Our findings differ from a recent study of telehealth use prior to the COVID-19 pandemic, which demonstrated lower telehealth use with increasing age.^17^ In our population, patients were more likely to utilize telehealth visits with increasing age during COVID-19 telehealth expansion, suggesting that the COVID-19 pandemic might have alter previous patterns of telehealth uptake. Higher rates of telehealth may be due to greater fear of contracting COVID-19 among older persons and the subsequent desire to quarantine and maintain social distance. Despite higher rates of telehealth visits, older patients were less likely to utilize full audio-video visits. A full audio-video telehealth visit requires a computer or mobile device and willingness and ability to download and use new software. Older patients are less likely to access the internet^20^ and use smartphones^21^ and health-information technology,^22^ likely contributing to higher rates of audio-only encounters.

Race and ethnicity are known to have a significant impact on COVID-19 incidence and mortality.^23^ In work done prior to the COVID-19 pandemic, large studies evaluating the relationship between telehealth use and race have yielded conflicting results.^14,17^ In our work, Black patients represented a greater proportion of encounters during the COVID-19 telehealth expansion compared to the same period prior year. During those COVID-19 period encounters, Black patients were less likely to use telehealth, and when telehealth was used, it was more often limited to audio capabilities alone. Despite previously conflicting evidence, our work suggests significant racial disparities in telehealth use during the COVID-19 PHE. These disparities may lead to worsening of existing racial disparities for health outcomes among racial and ethnic minority populations.

Surprisingly, rural residents were less likely to engage in telehealth encounters in our study, but more likely to use full audio-video capability. Rural residents have fewer healthcare services, fewer trained physicians and worse broadband coverage.^24^ None of these known associations explains our findings in the first 30 days of the COVID-19 telehealth expansion. Further research is needed to clarify the relationships between rural residence, distance from services, broadband coverage and telehealth use during the COVID-19 pandemic.

Payer status serves as a proxy for socioeconomic status^25^ and previous studies have associated low socioeconomic status with lower healthcare utilization.^26,27^ Our findings were mixed in this regard. Compared to the prior year, Medicaid and self-pay patients were less likely to reduce their healthcare utilization during the public-health emergency, and encounters for patients without insurance actually increased. The significance of and reasons for this finding are unclear but may reflect less perceived capacity to self-manage health conditions during the early phases of the pandemic. During the COVID-19 telehealth expansion, compared to patients with private insurance, patients with government payers and self-pay status had relatively higher odds of a telehealth encounter. It is not surprising that, compared to those covered by private insurance, telehealth encounters were more likely to be audio-only among those with government payer or with self-pay status, as those patients may be less likely to have the mobile devices and computers required for full audio/video telehealth.

Taken together, our findings reflect disparities in patients’ responses to the COVID-19 PHE. Telehealth is clinically effective and satisfying for patients to use. Further research should focus on the effect of these changes and disparities on downstream health outcomes, patient satisfaction, healthcare costs and interventions which might address inequities. Additional focused, community-engaged interventions may be needed to ensure equitable access to telehealth services in primary care settings during the COVID-19 PHE.

Our findings also have important implications for reimbursement policy in the USA. Initial telehealth reimbursement rates for telephone visits were substantially lower than for full audio-video telehealth encounters. While policy changes later rectified these inequities,^28^ providers who care for a disproportionate number of older, Black, Medicaid, or self-pay patients would have been disadvantaged financially. An increase in the use of telehealth will likely persist after the resolution of the COVID-19 pandemic,^29^ so reimbursement policies in the post-emergency period will need to account for these disparities.

Our study should be interpreted in the context of several limitations. Our study was performed during a disruptive period for patients and providers and may not reflect longer term patterns of telehealth use. This population of patients in central Missouri may underrepresent certain race, ethnicity and socioeconomic groups. ‘Rural’ as defined by FORHP postal code data and used in this study does not fully represent the spectrum of rurality in the USA. We only examined patient encounters and did not include appointments that were cancelled or not kept. A more complete portrait of the COVID-19 telehealth expansion would account for this activity. The chronic medical conditions for each patient were not evaluated and this is potentially a significant confounder. Finally, this study did not address clinical outcomes following telehealth visits, which is an important area for future research.

## Conclusion

Disparities exist in telehealth use by age, race, residence and payer in the period of telehealth expansion at the onset of the COVID-19 PHE. Further work is needed to clarify underlying causes these disparities and to inform policy-making during the COVID-19 emergency and beyond.
